# VCO_2_ calorimetry is a convenient method for improved assessment of energy expenditure in the intensive care unit

**DOI:** 10.1186/s13054-016-1397-z

**Published:** 2016-08-05

**Authors:** Ulrike Pielmeier, Steen Andreassen

**Affiliations:** Center for Model-based Medical Decision Support, Aalborg University, Fredrik Bajers Vej 7 E4, DK-9220 Aalborg Ø, Denmark

In their interesting article, Stapel et al. [1] suggested the use of carbon dioxide production (VCO_2_) calorimetry with energy expenditure (EE; kcal/day), calculated as 8.19 × VCO_2_ (ml/min), where VCO_2_ is provided by the built-in capnometer of the mechanical ventilator. This calculation on average overestimated EE by 7.7 % compared to indirect calorimetry (IC) with a standard deviation (SD) of ±8.4 %. This is within the ±10 % limits of acceptance used in many studies [2] and, more importantly, is an improvement relative to calculation of EE by predictive equations from the patient’s anthropometric data. The equation used by Stapel et al. incorporated a cohort respiratory quotient (RQ) of 0.86.

In his commentary, Pierre Singer [3] questions the usability of VCO_2_ for assessing EE in critically ill patients for three reasons: 1) the concept involves complicated mathematics; 2) calculation of RQ from the patient’s nutrition is complicated and uncertain; and 3) this invalidates the use of VCO_2_ calorimetry in the critically ill patient. We disagree with the first reason. Multiplying VCO_2_ by 8.19 is not complicated. We agree with the second reason. We unexpectedly saw significantly lower RQs in patients on a glucose-only diet compared with patients on enteral nutrition, such that individual RQ estimates calculated from the nutrition would have been inaccurate [4]. This does not imply that we agree with the third reason. In our sensitivity analysis we showed that changing our mean cohort RQ of 0.81 to 0.76, which is the lower end of the published range, only increased the VCO_2_ calorimetry estimates of EE by 6 %, while increasing RQ to the upper end of the published range, RQ = 0.89, reduced estimated EE by 8 %.

We recommended choosing a value of RQ = 0.85. With that choice, VCO_2_ calorimetry on average underestimated EE by 4 %, with an SD of 3 %, relative to EE estimated by IC [4], well within the ±10 % limits of acceptance. Our findings agree well with those by Stapel et al. and our conclusion is that VCO_2_ calorimetry is both easy and usable as a method for assessing EE for any cohort RQ within the published range (0.76–0.89).

The question remains whether VCO_2_ measured by built-in capnographs in various ventilators is sufficiently accurate. Stapel et al. found a 6.6 % systematic overestimation of VCO_2_ with their ventilator (SERVO-i; Maquet), compared to the gold standard (Deltatrac II; Datex). This is promising, but data are needed for other built-in capnographs.
