# Supplementary material for: VCO2 calorimetry is a convenient method for improved assessment of energy expenditure in the intensive care unit
Source: Crit Care. 2016 Aug 5;20:224. doi: 10.1186/s13054-016-1397-z (PMC4974728; doi:10.1186/s13054-016-1397-z)
Supplement: Related texts

# Reply: Author’s response

**Authors:** Sandra N. Stapel, Heleen M. Oudemans-van Straaten

We would like to thank Pielmeier and Andreassen for their valuable arguments in response to Pierre Singer’s commentary on our study about VCO_2_-derived measurement of EE.

In this study we concluded that EE can be accurately assessed at the bedside by multiplying ventilator-derived VCO_2_ by 8.19, especially when taking the mean 24-h value [1]. To calculate EE from ventilator-derived VCO_2_, we had to estimate the RQ in order to transform VCO_2_ into VO_2_. For study purpose, we used the RQ of nutritional intake, knowing there would be inaccuracy [5]. We actually found that measured RQ (0.859 ± 0.047) was quite similar to nutritional RQ (0.864 ± 0.015), but the two were not related at all (*p* = 0.485). In addition, the SD of the measured RQ was much larger. Thus, we share the opinion of both Pielmeier and Singer that calculating RQ from the administered nutrition is inaccurate. Nutritional RQ does not account for endogenous metabolism, which is unpredictable during critical illness. This was reported previously by McClave et al. [5] and is confirmed by the study of Rousing et al. [4], to which Pielmeier and Andreassen contributed.

Nevertheless, as we outlined, RQ only accounts for 15 % of the total bias of VCO_2_-derived EE. Since the mean RQ of our cohort and the RQ of most nutritional formulae are both 0.86, we proposed the simplified equation based on a fixed RQ of 0.86: EE = 8.19 × VCO_2_ (ml/min). In their interesting study, Rousing et al. [4] showed that, for any chosen RQ within the range of cohort values of 0.76 to 0.89, VCO_2_-based calorimetry performed significantly better than equations, thereby confirming our findings. They recommend using an RQ of 0.85, surprisingly similar to our conclusion.

Of note, IC remains the gold standard for assessment of EE in ventilated critically ill patients. However, the best-validated system, the Deltatrac, is no longer on the market and new indirect calorimeters have not yet proven to be accurate [6]. More importantly, predictive equations are inaccurate and their use should be avoided. VCO_2_-based EE provides the best alternative.

We agree with Pielmeier and Andreassen that we cannot extrapolate the results of our capnograph to other built-in capnographs. Their accuracy should first be validated, especially during irregular breathing. Furthermore, since we found a systematic error of 6.6 % for the VCO_2_ measurement, accuracy of the Maquet measurement should also be improved. The use of VCO_2_ measurements per second (instead of per minute) is currently under investigation.

## Abbreviations

EE, energy expenditure; IC, indirect calorimetry; RQ, respiratory quotient; SD, standard deviation; VCO_2_, carbon dioxide production
